# *Plasmodium falciparum* Pfs47 haplotype compatibility to *Anopheles gambiae* in Kisumu, a malaria-endemic region of Kenya

**DOI:** 10.1038/s41598-024-84847-6

**Published:** 2025-02-24

**Authors:** Shirley A. Onyango, Maxwell G. Machani, Kevin O. Ochwedo, Robin M. Oriango, Ming-Chieh Lee, Elizabeth Kokwaro, Yaw A. Afrane, Andrew K. Githeko, Daibin Zhong, Guiyun Yan

**Affiliations:** 1 School of Zoological Sciences, Kenyatta University, Nairobi, Kenya; 2 International Centre of Excellence for Malaria Research, Tom Mboya University, Homa Bay, Kenya; 3 Centre for Global Health Research, Kenya Medical Research Institute, Kisumu, Kenya; 4 Program in Public Health, College of Health Sciences, University of California at Irvine, Irvine, CA 92697 USA; 5 School of Biological Sciences, University of Nairobi, Nairobi, Kenya; 6 Department of Medical Microbiology, Medical School, University of Ghana, Accra, Ghana

**Keywords:** *P. falciparum*, Pfs47, *An. gambiae*, Compatibility, Geographic regions, Evolution, Genetics, Molecular biology

## Abstract

Insecticide resistance and outdoor transmission have reduced the effectiveness of existing malaria transmission prevention strategies. As a result, targeted approaches to support continuing malaria control, such as transmission-blocking vaccines, are required. Cross-sectional mass blood screening in children between 5 and 15 years was conducted in Chulaimbo, Kisumu, during the dry and wet seasons in 2018 and 2019. *Plasmodium falciparum* gametocyte carriers were identified by Microscopy. Subsequently, carriers were used to feed colony bred *Anopheles gambiae* females in serum replacement and whole blood membrane feeding experiments. The infection prevalence was 19.7% (95% Cl 0.003–0.007) with 95% of the infections being caused by *P. falciparum*. Of all confirmed *P. falciparum* infections, 16.9% were gametocytes. Thirty-seven paired experiments showed infection rates of 0.9% and 0.5% in the serum replacement and whole blood experiments, respectively, with no significant difference (P = 0.738). Six Pfs47 haplotypes were identified from 24 sequenced infectious blood samples: Hap_1 (E27D and L240I), Hap_2 (S98T); Hap_3 (E27D); Hap_4 (L240I); Hap_5 (E188D); and Hap_6 without mutations. Haplotype 4 had the highest frequency of 29.2% followed by Hap_3 and Hap_6 at 20.8% each then Hap_1 with a frequency of 16.7%, whereas Hap_5 and Hap_2 had frequencies of 8.3% and 4.2% respectively. Varying frequencies of Pfs47 haplotypes observed from genetically heterogeneous parasite populations in endemic regions illuminates vector compatibility to refracting* P. falciparum* using the hypothesized lock and key analogy. This acts as a bottleneck that increases the frequency of *P. falciparum* haplotypes that escape elimination by vector immune responses. The interaction can be used as a potential target for transmission blocking through a refractory host.

## Introduction

Insecticide resistance^1–4^ and outdoor transmission^5–8^ have compromised the efficacy of primary malaria control interventions, necessitating the development of new or improved targeted strategies that could complement the control of malaria, such as transmission-blocking approaches. Molecular mechanisms underlying *Plasmodium* infections and mosquito genotypes influencing parasite adaptations to diverse *Anopheles* species are critical in understanding malaria transmission dynamics and for developing targeted vector control interventions that may compliment already existing ones. 

Malaria transmission primarily depends on competent vectors and compatible infectious parasites to influence susceptibility in local *Anopheles* populations^9^. The mosquito immune factors, including recognition receptors, cellular and humoral components, influence the infectiousness of gametocytes in vectors^10^. The likelihood of infection after ingesting gametocytes from an infected person is determined by a combination of factors like the mosquito’s immune responses among others^10–13^. The thioester-containing protein 1 (TEP1) is an important immunological gene that exhibits allele-linked variations^14^ and also inhibits pathogens including *Plasmodium* infections in mosquitoes^15^, hence altering vector competence and malaria infectivity^16,17^. In contrast, the malaria parasite *P. falciparum* has evolved to circumvent the vectors’ immune responses mediated by the Pfs47^18,19^. The Pfs47 gene displays haplotypes that naturally select specific mosquito midgut receptors resulting in significant transmission variability^9^. According to Sinka et al.^20^, around 70 *Anopheles* species now transmit *P. falciparum* malaria around the world.

Recent studies have revealed that compatible Pfs47 haplotypes are selected by specific vector receptors in the midgut, eluding the immune system and increasing the likelihood of infection. However, incompatible haplotypes are detected and eliminated by the vector’s immune defenses^18^. Furthermore, selection pressures imposed by local *Anopheles* populations dominant in a given region may have altered the genetic diversity of Pfs47 haplotypes, resulting in parasite adaptations to native vector species. Therefore, the associations between the TEP1 immunity gene in *Anopheles* and Pfs47 in *P. falciparum* may be an important determinant of malaria infections and could be targeted in blocking malaria transmission in primary vectors efficient in transmitting malaria from a molecular perspective. Moreover, *Anopheles-Plasmodium* interactions are complex and have not been clearly understood yet, form a basis for increasing the knowledge gaps of host factors on vector competence. Also, these interactions are potential targets for developing malaria transmission-blocking interventions. The aim of this study is to determine parasite genotypes and their associations with mosquito infectivity in a malaria-endemic region in western Kenya.

## Materials and methods

### Study site and population

**Fig. 1:**
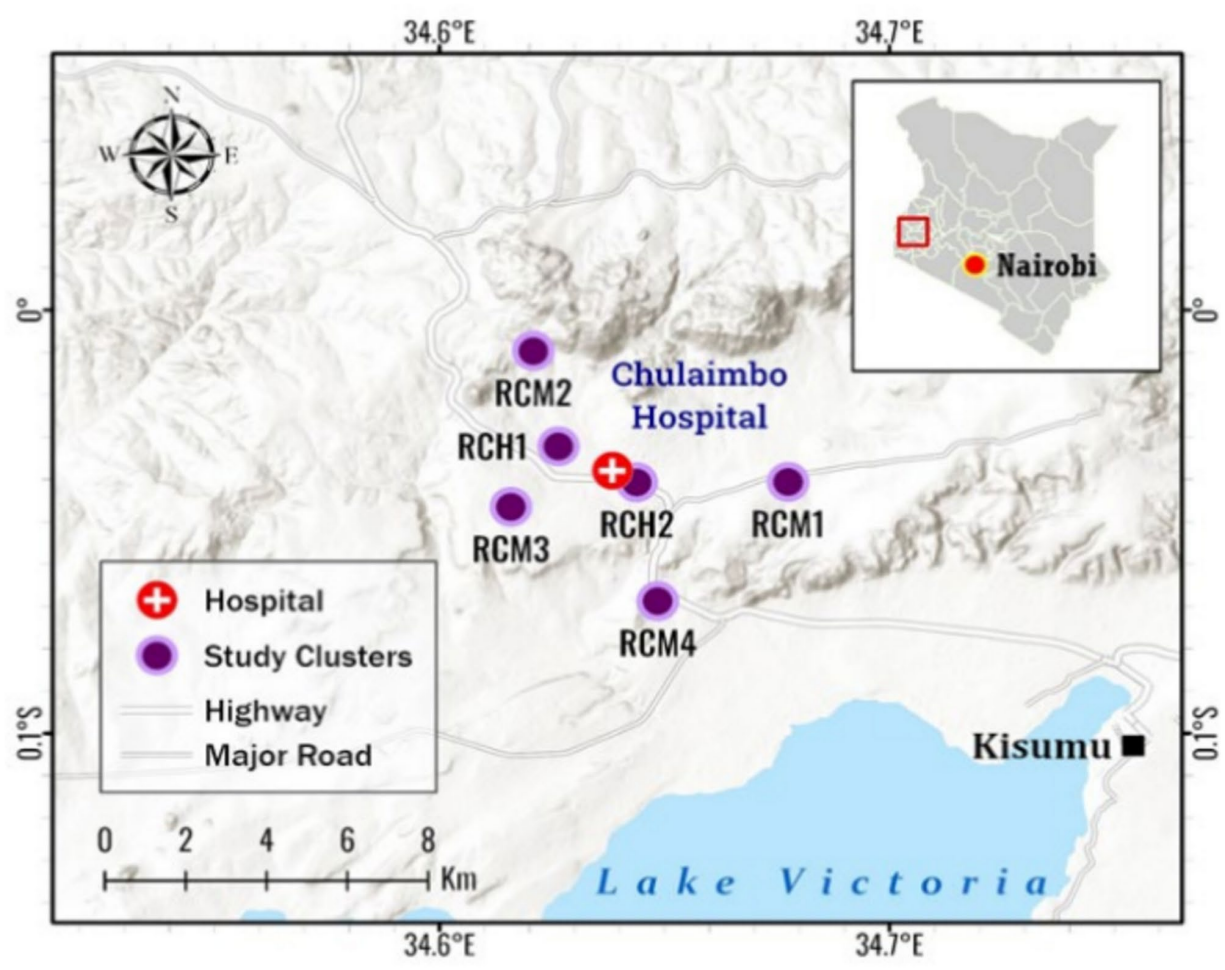
Map of Chulaimbo, Kisumu County showing the sampling locations. The map was generated using ArcGIS Pro 2.6 software. Map source: ESRI, CGIAR, and USGS (available at: www.esri.com).

Consented children aged 5–15 years were screened following a cross-sectional study design from sub locations in Chulaimbo, Kisumu County (Fig. 1), during the wet season from October to December 2019 and October to December 2020, and the dry season (January to March 2020). Site selection was purposive and based on an ongoing study of malaria prevalence and vector distribution. Chulaimbo is a rural site 19 km north-west of Kisumu City, located at 0.03572ºS, 34.621ºE, and an altitude range of 1328–1458 m above sea level^21^. The region has a mean annual temperature range of 12–35ºC. This region experiences an average annual rainfall of 1352 mm and an average relative humidity between 66 and 83%. Malaria transmission in this area is endemic, with *P. falciparum* as the dominant parasite species in the area^22^. Most residents are small-scale subsistence farmers.

### Mosquitoes used for the study

Laboratory reared *Anopheles gambiae* female mosquitoes (Kisumu strain) between 3 and 5 days post-emergence were used for membrane feeding assays. This colony was selected and maintained at the Center of Excellence for Malaria Research in Homa Bay, Western Kenya. They were reared at temperatures of 27–29 °C, 69–80% relative humidity (RH), and a 12 h light and 12 h dark cycle. The colony was then constantly maintained on 10% sucrose^23^ after the blood meal until the dissection day.

### Identifying gametocyte carriers

Parasitological assessments to detect *P. falciparum* gametocyte carriers were conducted in school-aged children from 5 and 15 years old, who had assented and had their guardians’ consent to participate in the study. Blood samples were obtained from the children using finger pricks on well-labeled Whatman® 903 Protein Saver Cards (GE Healthcare WB100014) with the participants’ information. A total of 50 µl of blood was collected onto the cards, air dried and stored at − 20 °C for further molecular analyses. Thick and thin smears were also prepared for the same participant stained with 10% Giemsa and read after drying. Parasites were viewed under a compound microscope and *Plasmodium* species identified in thick smears Malaria parasites counts were read against 500 white blood cells. Gametocyte densities were determined in slides for all *P. falciparum* positive participants by counting the number of gametocytes per 500 leukocytes by microscopy and expressed as parasites per μl assuming a standard white blood cell (WBC) concentration of 8000/μl^24^. Two trained microscopists took two readings per slide smear, and 20% of the slides were randomly selected for quality control verification by a senior external microscopist. Membrane feeding was limited to slides of gametocyte positive subjects only. Individuals who tested positive for malaria and had symptoms were referred to a local health center and treated according to Ministry of Health recommendations^25^.

### Mosquito infections using membrane feeding assays

Participants’ blood positive for gametocytes was used to infect insectary reared *An. gambiae* mosquitoes using serum replacement and whole blood experiments in the laboratory^26^. Blood was drawn intravenously by a professional phlebotomist using butterfly needles. Approximately 3 ml of blood was collected by venipuncture in heparinized tubes for each volunteer. An aliquot of 1 ml of blood was immediately placed into pre-warmed hemotek feeders (1 ml capacity) at 37 °C, while another 1 ml was transferred into 1 ml Eppendorf tubes and centrifuged at 2000 rounds per minute for 2 min before adding it to the hemotek feeders. The supernatant of serum was discarded and replaced with a naïve human serum type AB (Bio Whittaker, Cambrex Bio Science Walkersville, MD, USA). A final volume of 1 ml of replaced blood was then quickly transferred to the feeders to allow the starved mosquitoes to feed. Aggressive 3–5 days old female *An. gambiae* mosquitoes were starved for 6 to 8 h prior to feeding on infected blood. Whole blood and serum replacement experiments were conducted for each participant. A total of 37 paired experiments were conducted. Each feeding cup contained approximately 100–120 mosquitoes. The mosquitoes were allowed to feed from different feeders of the same infected blood for 15–30 min through a parafilm membrane. All membrane feeding procedures were conducted at 37 °C using the hemotek system. Only fully engorged blood-fed mosquitoes were selected and maintained at 27–29 °C temperatures and 69–80% relative humidity following a 12 h light and 12 h dark cycle. They were given 10% sucrose for 9 days post-feeding and the ones that survived were dissected for midgut oocysts enumeration. The unfed and partially fed mosquitoes were discarded by freezing them for 15 min at − 20 °C. After membrane feeding, volunteers were treated with artemether-lumefantrine (Coartem®) according to the Ministry of Health guideline^25^.

### Oocysts counts

All fully engorged mosquitoes that survived on day 8 or 9 post-feeding were dissected under a dissecting microscope as described by Afrane et al.^27^. Briefly, each mosquito gut was carefully pulled out from the abdomen in 0.5% mercurochrome and allowed to stain for 10 min. The midguts were then examined for the presence of oocysts under a light microscope. The number of oocysts observed were counted and recorded per mosquito gut. The oocysts load was expressed as the number of oocysts per infected mosquito. Slides were read by a technician and confirmed by a second technician. The positive and negative slides were then re-read by a third technician for quality control.

### Molecular analysis of TEP1 in infected mosquito carcasses

Mosquito carcasses corresponding to their infected midguts were labeled and preserved for further molecular assays to determine TEP1 genotypes^28^. Briefly, TEP1 was genotyped using a nested PCR–RFLP targeting 892 base pairs for nest 1 and a final fragment length of 758 base pairs after nest 2. Both PCR reaction conditions were set as denaturation at 95 °C for 3 min, 35 cycles of 94 °C for 30 s, annealing at 55 °C for 30 s, extension at 72 °C for 30 s, and a final step at 72 °C for 6 min using Dream Taq Green Master Mix (Thermo Fisher Scientific). PCR products were then digested using restriction enzymes Bam HI, Hind III, or Bse NI (New England Biolabs Inc) according to the manufacturer’s instructions and the result analyzed on 2.5% agarose gel electrophoresis. The TEP1 allelic classes were determined by fragment size of restriction enzyme digestion. A subset was also randomly selected for the genotype confirmatory purposes by sequencing.

### Parasite DNA extraction and Pfs47 genotyping

The Chelex technique was used to obtain *P. falciparum* gametocytes DNA from the dried blood spots confirmed by microscopy^29^. As previously reported^30^, a multiplex real-time PCR (RT-PCR) was utilized to identify *Plasmodium* species. Pfs47 was genotyped using PCR and Sanger sequencing, as previously published^30^. Briefly, forward 5’ATGTGTATGGGAAGAATGATCAG3’ and reverse 5’ACAAGTTCATTCATATGCTAACATA3’ primers were used to amplify the coding region 1320 bp from the DNA of *P*. *falciparum* gametocyte positive samples. A final reaction volume of 12 μl was prepared by addition of 6 μl of Dream Taq Green PCR Master Mix (2X), 0.5 μl of each of the forward and reverse primer, 3 μl of double distilled PCR grade water, and 2 μl of sample DNA. The PCR conditions were set as follows; 95 °C for 3 min, 35X (94 °C for30 sec, 50 °C for 30 s, 68 °C for 90 s), and 72 °C for 6 min before sequence, amplicons quality and size were determined by visualization of PCR products in 1.5% w/v gel under UV transilluminator. The amplicons were cleaned and sequenced directly using BigDye terminator chemistry v3.1, PCR primers, and PRISM® 3730xl genetic analyzer (Applied Biosystems, CA, USA). Paired reads from the sequencer were edited and assembled using BioEdit software (version 7.2.5) before further analysis.

### Ethical approval

The ethical review board of the Maseno University, Kenya (MSU/DRPI/MUERC/00456/17) reviewed and approved the protocol for screening of *P. falciparum* gametocyte carriers and subsequent intravenous blood drawing. A detailed written informed assent and consent to participate in the study was provided by all study volunteers and their parents or guardians. Feeding of mosquitoes was conducted in a secure, insect-proof room at the Chulaimbo health center. All experiments and methods were performed in accordance with the institution’s guidelines and regulations.

### Statistical analysis

Data from the participants was tabulated in Microsoft Excel V16. Computing descriptive statistics (sum, mean, standard deviation, standard error, and 95% confidence interval) and comparing means were done using Graph Pad Prism v.8.0.1 and SPSS version 25 for Windows software. The Shapiro–Wilk normality test was used to check data normality before performing pairwise comparisons and chi-square tests. Data were considered statistically significant at P < 0.05. The Codoncode Aligner 11.0.1 (CodonCode Corp., Centerville, MA) was used to check the sequence quality and trim low-quality bases. DnaSP version 6 software^31^ was used to infer haplotypes for samples with mixed infections, as indicated by heterozygous peaks in sequencing chromatograms. Bio-Edit software was used to align the sequences and determine the nonsynonymous mutations and codon changes based on reference sequence (Pf3D7_1346800). MEGA software was used to construct the UPGMA (unweighted pair group method with arithmetic mean) tree based on the Kimura 2-parameter (K2P) distance model with 1,000 bootstrap replicates.

## Results

### Parasitological surveys

A total of 4481 children were screened for malaria, 885 tested positive, representing a 19.7% infection prevalence (95% CI 17.9–21.5%). Most positive cases were attributed to *P. falciparum* infections, accounting for 95% (841) of the total infections. Other *Plasmodium* species identified in the study area were *P*. *malariae* (1.6%), *P. ovale* (0.3%), and mixed infections involving *P. falciparum* and *P. malariae* (*Pf/Pm*) or *P. ovale* (*Pf/Po*), each accounting for 2.7% of the infections. The gametocyte prevalence was 16.9% (142/841) with a 95% confidence interval (CI) ranging from 14.4 to 19.4% whereas the overall gametocyte density was 37.3 gametocytes/ µl of blood. The gametocyte density ranged from 16–176 gametocytes/ µl of blood treating each infection as an individual entity. The odds of finding microscopic gametocyte infections were significantly high during the dry season (OR 1.37, 95% CI 1.14 to 1.64, P = 0.001) compared to the wet season. The males were more likely to harbor microscopic gametocyte infections (OR 1.23, 95% CI 1.06 to 1.42) than females (Supplementary Table 1).

### Mosquito infections

Out of 142 children who tested positive for *P. falciparum* gametocytes, 37 children were each subjected to both serum replacement and whole blood membrane feeding experiments. Only 24% (9/37) of the children successfully infected one or more mosquitoes 9 days post-feeding. A total of 3894 mosquitoes were dissected, 1960 in serum replacement and 1934 in whole blood to evaluate infection rates. The paired experiments were conducted from the same donor. The infection rates were 0.8% (15/1960) in serum replacement and 0.5% (9/1934) in whole blood whereas the oocyst densities were 1 and 1.8 respectively (Supplementary Table 2). However, the difference in infection rates between the two experiments was not statistically significant (P = 0.738). All *An. gambiae s. s* mosquitoes (n = 205) utilized were from a susceptible strain reared in the insectary and harbored the homozygous TEP1* S1/S1 genotypes.

### *Plasmodium falciparum* Pfs47 haplotypes

**Fig. 2:**
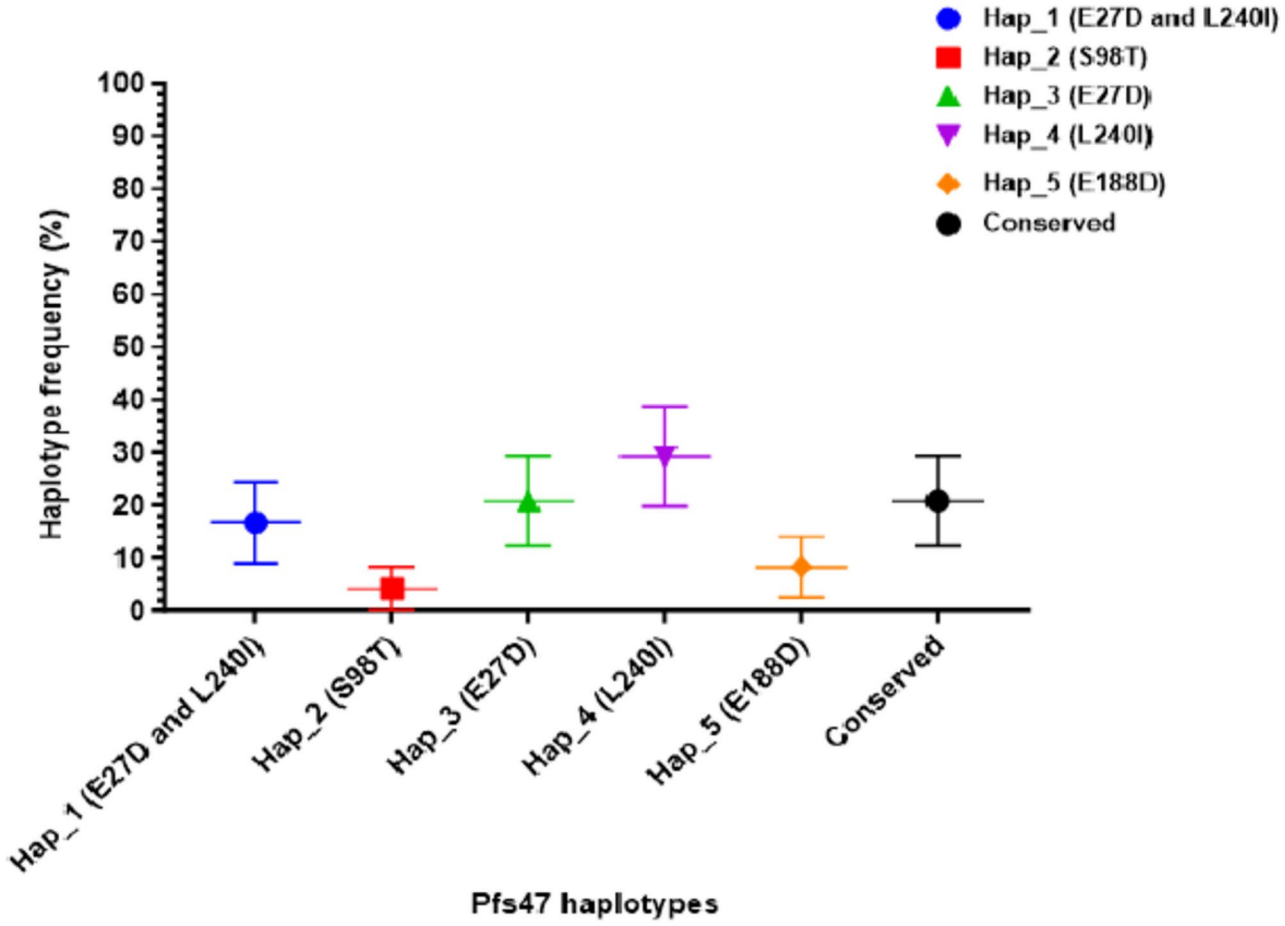
Frequency of infectious Pfs47 haplotypes in *Plasmodium falciparum*.

Six haplotypes were identified from the sequenced gametocyte containing dried blood spots (DBS) confirmed by microscopy. Haplotype 1 (Hap_1) had dimorphic codon E27D and L240I, Hap_2, Hap_3, Hap_4, Hap_5 had S98T, E27D, L240I, and E188D mutations respectively, whereas, Hap_6 was conserved or had no polymorphic site (Fig. 2). Genotyped parasite DNA from blood with Hap_4 with the dimorphic codon L2401 was frequent at 29.2% (7) with positive oocyst results followed by Hap_3 (E27D) and Hap_6, each with 20.8% (5). haplotypes with E27D and L240I mutations (Hap_1) were at a frequency of 16.7% (4) whereas Hap_2 (S98T) and Hap_5 (E188D) was each present at a frequency of 4.2% (1) and 8.3% (2) respectively.

## Discussion

Malaria vector and parasite interaction is a key determinant towards successful transmission^9,32^. For an established or localized transmission system, there is need for susceptible vector genotype populations and infectious *Plasmodium* haplotypes that evoke endogenous compatible immune evasive elements that circumvent them. This study shows the compatibility of *Pfs47* haplotypes characterized by specific codon variants that may influence infectivity to *An. gambiae* mosquitoes. These results may have implications on the parasites capacity to evade the vectors’ immune defenses effectively completing its transmission cycle. Haplotype 4 with codon L240I had the highest frequency among the haplotypes that progressed to detectable oocyst in the midgut and could be linked to increased infectivity or transmission potential to vectors followed by haplotypes; Hap_3 (E27D) and Hap_6 (without polymorphic sites) then Hap_1(E27D and L240I). Despite Hap_1 having two dimorphic sites E27D and L240I, the genotypic combination did not appear to increase its frequency or malaria transmission in the region. The other haplotypes Hap_2(S98T) and Hap_5(E188D) also infected mosquitoes displaying a probable limited compatibility. Although this study used DBS for genotyping Pfs47, genotyping the positive midguts would provide a more definitive link to observed phenotypes to a particular haplotype. The compatibility of the six haplotypes should therefore be evaluated further using field collected *Anopheles* mosquitoes which are likely to possess heterogenous TEP1 alleles providing critical insights into the transmission dynamics of *P. falciparum* in natural settings.

A previous study conducted from western Kenya identified thirteen Pfs47 haplotypes, with haplotypes harboring the mutation codon E27D having the highest frequency of over 50%, followed by conserved Pfs47 haplotypes whereas the rest occurred at a frequency of 6.7% or lower^30^. Even though haplotypes with E27D were most common, haplotypes with L240I were more infectious to *An. gambiae s. s.* which may imply that it has enhanced evasion of the vector immune defenses. The TEP1* S1/S1 allele observed in mosquitoes used for this study is an indication of populations being susceptible to *Plasmodium* gametocytes^33,34^ and may have been highly likely compatible with gametocytes containing the L240I dimorphism following the “lock-and-key” analogy described by Molina-Cruz et al.^9^ unlike the other infectious haplotypes that were identified in the region. Despite this, mosquito populations reared in laboratory settings are susceptible to inbreeding depression and can adapt to artificial conditions that differ significantly from those in the field.

The *Pfs47* gene has undergone natural selection as a result of adaptations to diverse anopheline species found in different continents hence a strong population structure^9,32,35^. Furthermore, parasites with compatible Pfs47 haplotypes can elude complement activation and survive within invaded midgut cells^36^. To evaluate the possible influence on the dynamics of malaria transmission, it is essential to understand the frequency and distribution of these haplotypes. Higher transmission rates might result from some haplotypes’ greater ability to overcome mosquito immune responses. Apart from vectors and gametocyte compatibility human antibodies against gametocytes play a critical role in transmission blocking or reduction capabilities in addition to their compatibility^37–39^. As a result, high gametocyte densities (> 20 gametocytes/µl) may not always indicate successful mosquito infection. Conversely, low gametocyte densities (< 10 gametocytes/µl) do not necessarily exclude the possibility of infectiousness^40,41^. A weak association between gametocyte density and infection rates was observed despite exposing numerous mosquitoes with infected blood. The low infection rates may have been due to other factors including the sex ratios of the gametocytes, the genetic makeup of the gametocytes, and the immune factors may have inhibited infections in the mosquito. This finding corroborated previous investigations^42^ that also documented low infection rates, and weak association between gametocyte densities and mosquito infection rates which varied with low gametocyte densities. This study however, may have underestimated the gametocyte prevalence due to the presence of submicroscopic infections detectable by PCR.

## Conclusion

The study highlights the critical role of vector and parasite interaction in malaria transmission, emphasizing the need for susceptible vector genotypes and infectious *Plasmodium* haplotypes. The findings show that specific Pfs47 haplotypes, particularly those with the L240I codon variant, are more compatible with *An. gambiae* mosquitoes, suggesting enhanced immune evasion. Haplotype 4 (Hap_4) with the L240I variant exhibited the highest frequency and potential for infectivity, followed by other haplotypes. The study also underscores the importance of natural selection and adaptation of the Pfs47 gene, which may influence transmission dynamics. The findings suggest that targeting Pfs47 haplotypes linked to increased mosquito infection rates could be a potent approach for controlling malaria.

## Supplementary Information

Supplementary Tables.

## Data Availability

The data supporting the conclusions of this study is included within the article and its supplementary information files.
