# Supplementary material for: Plasmodium falciparum Pfs47 haplotype compatibility to Anopheles gambiae in Kisumu, a malaria-endemic region of Kenya
Source: Sci Rep. 2025 Feb 24;15:6550. doi: 10.1038/s41598-024-84847-6 (PMC11850800; doi:10.1038/s41598-024-84847-6)
Supplement: Supplementary file 1 — Supplementary Tables. [file 41598_2024_84847_MOESM1_ESM.docx]

**Supplementary Table 1: Gametocyte density and prevalence by season and gender in Kisumu**

| **Parameter** | | **Number screened** | **Gametocyte Density/µl** | **Gametocyte Prevalence**  **n (%)** | **χ^2^** | **P value** | **OR (95% CI)** |
| --- | --- | --- | --- | --- | --- | --- | --- |
| **Season** | Wet | 3690 | 30.88 (26.67 - 35.74) | 682 (18.5) | 11.09 | 0.001 | 1.37 (1.14-1.64) |
|  | Dry | 791* | 44.11(28.43 - 68.42) | 187 (23.6) |  |  |  |
| **Gender** | Female | 2262 | 32.03 (26.15 -39.23) | 403 (17.8) | 7.27 | 0.007 | 1.23 (1.06-1.42) |
|  | Male | 2219* | 31.97 (26.30 - 38.84) | 466 (21) |  |  |  |

*Reference categories

**Supplementary Table 2 Infection prevalence for paired experiments (n=37)**

| **Experiment types** | **Number of mosquitoes exposed** | **Feeding rate (%)** | **Number of mosquitoes Dissected (%)** | **Number of mosquitoes Infected** | **Prevalence of infection (%)** | **Total oocysts count** | **Oocyst density/midgut** |
| --- | --- | --- | --- | --- | --- | --- | --- |
| Serum replacement | 3760 | 65.20% | 1960 (82.2) | 15 | 0.8 | 15 | 1 |
| Whole blood | 3760 | 56.60% | 1934 (80.2) | 9 | 0.5 | 16 | 1.8 |
